# Uptake of Non-Mandatory Vaccinations in Future Physicians in Italy

**DOI:** 10.3390/vaccines9091035

**Published:** 2021-09-17

**Authors:** Chiara Bertoncello, Annamaria Nicolli, Stefano Maso, Marco Fonzo, Mariaangiola Crivellaro, Paola Mason, Andrea Trevisan

**Affiliations:** Department of Cardiac Thoracic Vascular Sciences and Public Health, University of Padova, via Giustiniani 2, 35128 Padova, Italy; chiara.bertoncello@unipd.it (C.B.); annamaria.nicolli@unipd.it (A.N.); stefano.maso@unipd.it (S.M.); marco.fonzo@unipd.it (M.F.); mariaangiola.crivellaro@aopd.veneto.it (M.C.); paola.mason.1@unipd.it (P.M.)

**Keywords:** non mandatory vaccination, mandatory vaccination, future physicians, vaccination compliance

## Abstract

In 2017 in Italy, a number of vaccinations became mandatory or started to be recommended and offered free of charge. In this study, we aimed at assessing the coverage rates for those vaccinations in the pre-mandatory era among students at the School of Medicine of Padua University studying the degree course in medicine and surgery (future physicians) on the basis of the vaccination certificates presented during health surveillance. The vaccinations considered were those against pertussis, rubella, mumps, measles, varicella, *Haemophilus influenzae* type b (which became mandatory in 2017), pneumococcus, meningococcus C and meningococcus B (only suggested and offered for free since 2017). The study enrolled 4706 students of medicine and surgery. High vaccine uptake was observed, especially in younger students (born after 1990), with vaccines against pertussis, rubella, mumps and measles. Good completion for *Haemophilus influenzae* type b and meningococcus C was also observed. Very low coverage rates (all under 10%) for vaccination against varicella, pneumococcus and meningococcus B were observed. In conclusion, uptake for some non-mandatory vaccines was below the recommended threshold, although younger generations showed a higher uptake, possibly as a results of policy implemented at the national level. Our findings support the idea to consider health surveillance visits also as an additional opportunity to overcome confidence and convenience barriers and offer vaccine administration.

## 1. Introduction

The history of mandatory vaccinations in Italy begins in 22 December, 1888 with the law N. 5849 [1], which implemented mandatory vaccination against smallpox (reaffirmed by the Royal Decree July 27, 1934, N. 1265 [2] for all newborns within the first year of life); the vaccination was subsequently suspended in 1977 and revoked in 1981. Successively, the Decree of the Head of Government (2 December 1926) implemented mandatory vaccination against typhus [3] for kitchen staff and others (the first mandatory vaccination for workers), which was then abolished in 1997 and revoked in 2000. Furthermore, the law of 6 June 1939, number 891, introduced mandatory vaccination against diphtheria [4]. In the 1960s, tetanus vaccination was introduced (law 5 March 1963, N. 292) [5], initially for various categories of at-risk workers (the only example of mandatory vaccination for workers still existing in Italy until 2021), followed by vaccination against poliomyelitis (law 4 February 1966, N. 51) [6].

In 1991, mandatory vaccination against hepatitis B was implemented (law 27 May 1991, N. 165) for all newborns and adolescents in the twelfth year of life [7].

As a consequence of the measles outbreak in 2017, the legislative decree of 7 June 2017, N. 73, added mandatory vaccination against pertussis, varicella, rubella, mumps, measles and *Haemophilus influenzae* type b (Hib) up to 16 years of life to the list of existing mandatory vaccinations (diphtheria, tetanus, poliomyelitis and hepatitis B) [8]. Up until 1 April 2021, there were ten mandatory vaccinations in Italy.

The SARS-CoV-2 (COVID-19) pandemic proposed an unexpected scenario and, in order to cope with the hesitancy of some healthcare workers towards the vaccine, the Italian government was forced to enact a law related to mandatory vaccinations reserved for these professionals (law decree 1 April 2021, N. 44) [9]. Table 1 summarises all mandatory vaccinations (valid or revoked) implemented in Italy from 1888 to the present day.

At this moment, there are ten mandatory vaccinations for newborns (diphtheria, tetanus, poliomyelitis, hepatitis B, pertussis, varicella, rubella, mumps, measles and Hib) and two mandatory vaccinations for specific categories of workers (tetanus and COVID-19).

This is the current state of mandatory vaccinations by law, but we must also consider non-mandatory vaccinations. For this purpose, this research was conducted to highlight the completion of a whole series of non-mandatory vaccinations (Table 2) implemented in a pre-mandatory era, both administered in childhood and adolescence.

The aim of the present research is to explore, based on the vaccination certificates released by the Public Health Office, the completion of non-mandatory vaccination in a population of students attending the medicine and surgery degree courses of the University of Padua (Northeastern Italy) in the pre-mandatory era.

## 2. Materials and Methods

### 2.1. Population

Data were collected on a population of 4706 students at the School of Medicine of Padua University (degree course in medicine and surgery), collected as part of health surveillance according to the law from 2007 to 2020. There were 2632 females and 2074 males (ratio 1.27) selected. Participants were only eligible if they were born from 1980 onwards. The year 1980 was selected because students born after this year were subjected to all four mandatory vaccinations available at the time and, obviously, were not subjected to the further mandatory vaccinations implemented by law in 2017 [8]. Students were enrolled only if a certificate released by the Public Health Office was available and if they were born in Italy; they were further divided into five year of birth groups (born between 1980 and 1985, 1986 and 1990, 1991 and 1995 and after 1995).

Among the non-mandatory vaccinations, we considered those that were subsequently indicated as mandatory by Legislative Decree 73/2017 [8], which are now common vaccinations administered during infancy, together with mandatory vaccines against pertussis, rubella, mumps, measles, varicella, Hib and those suggested by the same decree and provided free of charge, such as those against pneumococcus (polysaccharide), meningococcus C (Men C) and meningococcus B (Men B). The Legislative Decree also suggests vaccination against rotavirus, but since it is recommended to be administered at less than six months of life, and because it was implemented in Europe in 2006, it was not taken into consideration.

### 2.2. Statistics

The 2 by 2 (Yates’ correction) chi-square (χ^2^) test was used to compare the percentage of vaccination compliance. Significance was indicated by a *p*-value of <0.05. Statsdirect 2.7.7 version (Statsdirect Ltd., Birkenhead, Merseyside, UK) was used for the statistical analyses.

## 3. Results

**Figure 1:**
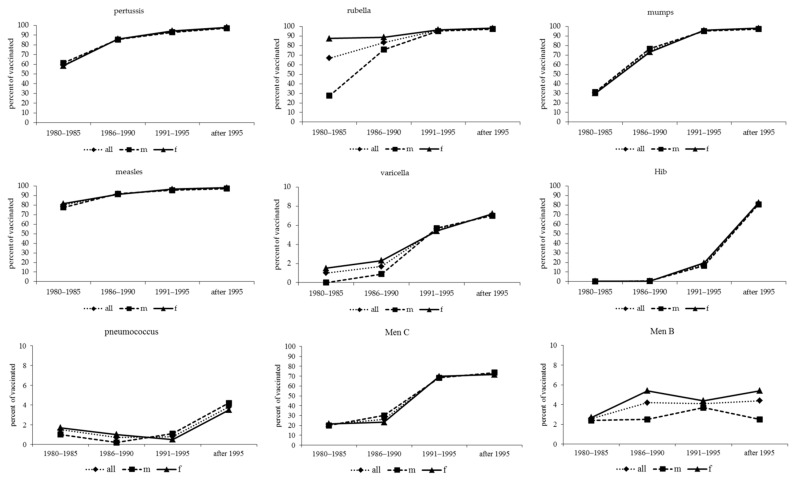
Compliance with vaccination against pertussis, rubella, mumps, measles, varicella, Hib, pneumococcus, Men C and Men B in pre-mandatory era. These vaccinations became mandatory for all newborns and those up to 16 years of age in 2017, except those against pneumococcus, Men C and Men B.

Coverage rates for non-mandatory vaccinations against common childhood infectious diseases, such as pertussis, rubella, mumps, measles, varicella, Hib, pneumococcus, Men C and Men B (Figure 1) are different according to the participants’ year of birth. Vaccination coverage rates for rubella, mumps and measles, consistently increased, especially in the beyond 1990 year of birth groups. In relation to sex, coverage rates for pertussis, measles, varicella, Hib and pneumococcus are similar. Against mumps, males are significantly more likely to be vaccinated than females (*p* = 0.0002), but only in total and not according to the year of birth groups. Rubella is a vaccination with a high prevalence in females (*p* < 0.0001), but only in the first two year of birth groups (*p* < 0.0001). Students born after 1995 achieved a vaccination coverage of over 97% for pertussis, rubella, mumps and measles and near 80% for Hib. No sex-related differences were observed for vaccinations against pneumococcus, but, in any case, compliance with this vaccine was 1.7% overall. On the contrary, a consistent percentage of students was vaccinated against Men C (higher than 50%), especially the cohort born after 1995, which exceeded 70% (72.4%). The compliance of males was significantly (*p* = 0.0023) higher than that of females. Finally, coverage rates for vaccination against Men B were low (4.0%); in this case, females were significantly (*p* = 0.0093) more compliant than males. Overall date are summarized in Table 3.

## 4. Discussion

Our study showed that uptake for non-mandatory vaccinations in future physicians varies greatly, ranging from 93.5% for measles to 1.7% for anti-pneumococcal vaccination. This variability, although large, seems to reflect the vaccination coverage levels in the general population in Italy, its temporal trends and possibly the effect of vaccination policies at the national level. Overall, rather than the mere proportion of vaccinated individuals, it is interesting to consider temporal trends by participants’ age (Figure 1): for all vaccines included in the study a stabilisation or an increase over time was noted. For instance, vaccination coverage rates for rubella, mumps and measles consistently increased over time, especially in individuals born after 1990. This may be due to the introduction of the MMR formulation in 1999 [17], which may have contributed to the rise in the coverage rate and to maintain it close—but not above, as recommended—to the threshold of 95% [19]. The completion of rubella vaccination is significantly higher in females than males (93.8% and 84.8%, *p* < 0.0001, respectively); this discrepancy may be attributable to two major factors: (1) the awareness in the general population that women of child-bearing age are at a high risk of foetal rubella syndrome in the event of infection and (2) the active on-site offering of rubella vaccinations for teenage females during the last year of primary and the first year of secondary school since 1972 to the 1988–1989 cohort. Very low levels for vaccination against Men B may be mainly due to the fact that implementation began only in 2013. In a similar manner, coverage rates for varicella vaccine stand at 4.5%, and the reason behind this may be that the implementation occurred only in 2001, so that only the youngest participants in our study may have received the varicella vaccine.

Strategies aimed at increasing vaccination coverage are varied. Evidence showed that a number of different intervention strategies have been taken into account, as for instance, educational interventions aimed at improving awareness and perception of both the disease and the vaccine availability [20,21], health literacy interventions [22], facilitation of healthcare access and delivery [23,24] and as a last resort whenever needed, the establishment of compulsory vaccination.

The recommendation for non-mandatory vaccinations favoured a decrease in several infectious diseases over time, but an improvident “loss of confidence”, with the growth of the anti-vax movement and vaccination hesitancy causing a progressive decline in vaccine coverage in Italy [25] has to be considered. This decline was probably the main cause of the measles outbreak in 2017. The measles epidemic of 2017 in Italy prompted the government to enact a law to make 10 vaccines, including measles, mandatory from children aged 0 to 16 years [8]. A similar scenario happened in France, where a gradual decline in vaccine uptake alongside an increase in measles incidence prompted the government to include eight more vaccines in the paediatric immunisation schedule, along with diphtheria, tetanus and polio vaccines that were already mandatory; the law went into effect in 2018. On the other hand, since 2015 in Germany, in order to have their child go to school, parents must provide proof of a routine check-up, including an immunisation counselling session provided by a doctor. A fine of up to 2500 euros is expected for parents who do not attend the counselling. [26]. Evidence from a recent systematic review supports the contention that mandatory vaccination and the magnitude of fines are associated with higher vaccination coverage, and a lower incidence of measles has been noted in European countries with mandatory vaccination [27]. As an example, in France, a 2.6% increase in MMR uptake was noted, while hepatitis B vaccine coverage in infants increased by 5.5% a few months after the implementation of the law [26]. However, while the mandatory effect has resulted in a significant increase in vaccination coverage [28,29], this information is not always understood and shared.

Whether vaccinations should be mandatory or non-mandatory (the Shakespearean dilemma) is an open question in vaccination programs. Since 2017 [8], ten vaccines have been mandatory in Italy, i.e., those against diphtheria, tetanus, poliomyelitis, hepatitis B (already mandatory, and for this reason not considered in the present study) as well as those against rubella, mumps, measles, varicella, Hib and pertussis in the 0–16-year-old group. It should be remembered that none of these vaccines were specifically mandatory for healthcare workers (HCWs). Three requirements for mandatory vaccination, from an ethical perspective, are necessary: (1) The disease must be a serious threat to the health of children and to public health; (2) mandatory vaccination must present a positive comparative expected usefulness; and (3) coercion must be proportionate [30].

Making vaccination a legal requirement can be powerful to “handle with care” with regard to the context. Current evidence suggests that a mixed approach including punitive and flexible policies might be more effective [31]. As stated in an article by Bester, an important element in increasing adherence to vaccination is to rebuild the relationship of trust between the health professional and the person to be vaccinated, or the parents in the case of minors. If this trust is not re-established, which could be further eroded by coercive interventions, educational interventions will also fail [32].

The active offering of vaccines increases compliance and allows us to achieve good coverage, but mandatory vaccination appears sometimes necessary and diseases that require extensive coverage, such as measles would benefit to a greater extent from a regulatory intervention.

The risk perception, rather than the frequency of events, improves compliance [20] for example, varicella, Men C and Hib are actively offered, but we have always been more concerned about meningitis (which is more lethal and less frequent) than varicella. Obviously, the availability of combined vaccines also affects compliance (as in the case of pertussis and Hib). In fact, pertussis and Hib benefit from the fact that they are administered together with mandatory vaccines, such as MMR, which are administered at the same time or close to the former vaccinations.

In any case, the results of this survey show that vaccination against some infectious diseases, in particular pertussis, measles, mumps, rubella and most recently, Hib have a particularly high compliance rate although they did not reach the desired threshold (these vaccines have recently become mandatory). Moreover, Men C and pneumococcus (non-mandatory) vaccinations have also seen a significant increase in compliance since 2017 [33]. The level of compliance with rubella vaccination is interesting, and is mainly complied with by women in the first instance; then, when offered with measles and mumps vaccines, it is also complied with by the other sex. A similar trend could also be assumed for the HPV vaccine, which was initially intended only for women, but was then also offered to males in the knowledge that they can infect their female partners [21]. However, it will take a few years (perhaps another decade) to witness a phenomenon similar to that shown for rubella. Finally, the phenomenon of vaccine hesitancy is a cause of low vaccination coverage, even among HCWs [34,35]. As previously reported [36], this phenomenon will certainly be much less evident in the near future given that our data show a high level of vaccination coverage in those that will be future HCWs. Overall, there is a good response from future HCWs (and their parents) towards this type of vaccination. On the other hand, the percentage of HCWs vaccinated against varicella remains low, but all were born in the pre vaccination era and, in any case, at a time in which there was high viral circulation [37]. Furthermore, in the case of pertussis, the administration of a booster dose is almost always necessary because both disease-induced and vaccine-induced antibodies take a relatively short time to disappear [38,39,40].

## 5. Conclusions

The present research investigated the compliance of students enrolled in a degree course in medicine and surgery (therefore future physicians) with non-mandatory vaccinations at the time of their enrolment.

Overall, the coverage rates for non-mandatory vaccines are in line with vaccine uptake in the general population of same age with some vaccines below the recommended thresholds, although younger generations showed a higher vaccine uptake, possibly as a result of policies implemented at the national level. Our findings confirm the goodness of health surveillance protocols to include the determination of antibodies against the microorganisms that cause infectious transmissible diseases in order, if necessary, to administer a booster dose (or a full cycle) and support the idea to consider health surveillance visits for future physicians also as an additional opportunity to do health promotion, including an improvement in vaccine uptake.

## Figures and Tables

**Table 1:** History of mandatory vaccination in Italy.

Biological Agent	Law	Status
smallpox	Law 22 December 1888, N. 5849 [1]	suspended in 1977
	Royal Decree 27 July 1934, N. 1265 [2]	revoked in 1981
typhus	Decree of the Head of Government 2 December 1926 [3] Mandatory for kitchen staff, water supply services, milk collection and distribution services, hospital laundry, cleaning and disinfection services, recruits	abolished December 27, 1997 revoked 23 January 2000
diphtheria	Law 6 June 1939, N. 891 [4]	valid
tetanus	Law 5 March 1963, N. 292 [5] for all children in the second year of life and Mandatory for agricultural workers, shepherds, ranchers, grooms, jockeys, tanners, overseers and persons in charge of preparing the racetrack tracks, street sweepers, roadmen, roadsters, excavators, miners, bakers, construction workers and unskilled workers, railway workers and unskilled workers, asphalt planners, rag vendors, garbage handling workers, paper and cardboard manufacturing workers, wood workers, metallurgists and metalworkers. The vaccine is mandatory for all new recruits. The Ministry of Health is authorised to extend, with its own decree, the obligation of vaccination to other categories of workers, after consulting the Superior Health Council	valid
poliomyelitis	Law 4 February 1966, N. 51 [6]	valid
tuberculosis	Law 14 December 1970, N. 1088 [10] Mandatory for medical students enrolled from 1970 to 2001	suspended after 2001
hepatitis B	Law 27 May 1991, N. 165 [7] for all newborns (since 3rd month of age and adolescents in the 12th year of age)	valid
pertussis, varicella, rubella, mumps, measles and Hib	Legislative Decree 7 June 2017, N. 73 [8] For newborns and adolescents 0–16 years	valid
SARS-CoV-2 (COVID-19)	Law Decree 1 April 2021, N. 44 [9] Mandatory for those who work in health professions and health professionals who carry out their activities in public and private health, social and social welfare structures, pharmacies, parapharmacies and professional offices	valid

**Table 2:** Non mandatory vaccinations implemented in Italy according to different population groups and users. Measles, mumps, rubella (as MMR), varicella, pertussis and Hib became mandatory in 2017 according to a 2017 legislative decree [8]. The same legislative decree also recommends vaccination against meningococcus C (Men C), meningococcus B (Men B), pneumococcus and rotavirus in the same population 0–16 years of age; these vaccines are provided free of charge.

Vaccines	Year of Implementation	Doses (Basic Cycle)	Comments
Pertussis	Whole cell 1962, acellular 1995 [11]	5	Mandatory
Rubella *	1972 [12] **	2	Mandatory
Mumps *	1982 [13]	2	Mandatory
Measles *	1979 [14]	2	Mandatory
Hib	1999 [15]	3	Mandatory
Varicella	2001	2	Mandatory
Pneumococcus	2001 [16]	3	Suggested and free of charge
Men C	2005	3	Suggested and free of charge
Men B	2013	2	Suggested and free of charge
Rotavirus	2006	2/3	Suggested and free of charge

Legend: * measles, mumps and rubella vaccines are administered together as MMR according to their implementation in 1999 [17] and 2001 [18]. ** Vaccination was initially recommended for pre-pubescent girls only.

**Table 3:** Summary of the overall data on the nine vaccinations. Percentage of students vaccinated against pertussis, rubella, mumps, measles, varicella, Hib, pneumococcus, Men C and Men B in total and subdivided according to sex. Statistical significance is related to the comparison between males and females.

Vaccine	All N.	Vaccinated	%	Males N.	Vaccinated	%	Females N.	Vaccinated	%	*p*
Pertussis	4706	4169	88.6	2074	1853	89.3	2632	2316	88.0	=0.1614
Rubella	4706	4228	89.8	2074	1758	84.8	2632	2470	93.8	**<0.0001**
Mumps	4706	3910	83.1	2074	1771	85.4	2632	2139	81.3	**=0.0002**
Measles	4706	4401	93.5	2074	1938	93.4	2632	2463	93.6	=0.8973
Varicella	4706	214	4.5	2074	93	4.5	2632	121	4.6	=0.9088
Hib	4706	1408	29.9	2074	622	30.0	2632	786	29.9	=0.9502
Pneumococcus	4706	80	1.7	2074	37	1.8	2632	43	1.6	=0.7777
Men C	4706	2550	54.2	2074	1176	56.7	2632	1374	52.2	**=0.0023**
Men B	4706	188	4.0	2074	65	3.1	2632	123	4.7	**=0.0093**

Legend: Significant results are shown in bold.

## Data Availability

Raw data are available on request from the corresponding author.
